# The Effect of 10 Crop Plants That Served as Hosts on the Primary Metabolic Profile of the Parasitic Plant *Phelipanche aegyptiaca*

**DOI:** 10.3390/metabo12121195

**Published:** 2022-11-29

**Authors:** Krishna Kumar, Yael Hacham, Rachel Amir

**Affiliations:** 1 Migal–Galilee Technology Center, Tarshish 2, Kiryat Shmona 1101600, Israel; 2 Tel-Hai College, Upper Galilee 1220800, Israel

**Keywords:** different hosts, GC-MS analysis, holoparasitic plant, metabolic changes, *Phelipanche aegyptiaca*

## Abstract

*Phelipanche aegyptiaca* Pers. is a holoparasitic plant that parasitizes various types of host plants. Its penetration into host roots causes a massive reduction in the yield of many crop plants worldwide. The nature of the compounds taken by the parasite from its host is still under debate in the scientific literature. To gain more knowledge about the effect of the hosts on the parasite’s primary metabolic profile, GC-MS analyses were conducted on the parasites that developed on 10 hosts from four plant families. There are three hosts from each family: Brassicaceae, Apiaceae and Solanaceae and one host from Fabaceae. The results showed significant differences in the metabolic profiles of *P. aegyptiaca* collected from the different hosts, indicating that the parasites rely strongly on the host’s metabolites. Generally, we found that the parasites that developed on Brassicaceae and Fabaceae accumulated more amino acids than those developed on Apiaceae and Solanaceae that accumulated more sugars and organic acids. The contents of amino acids correlated positively with the total soluble proteins. However, the aromatic amino acid, tyrosine, correlated negatively with the accumulation of the total phenolic compounds. This study contributes to our knowledge of the metabolic relationship between host and parasite.

## 1. Introduction

The Orobanche and Phelipanche species are holoparasitic plants that lack chlorophyll. In Israel, there are five species that belong to this genus that attack agricultural crops, but the dominant and most common one is Egyptian broomrape (*Phelipanche aegyptiaca* Pers.) [1]. This parasite causes heavy damage and a significant reduction in the yield of crop plants around the world [2]. *P. aegyptiaca* attacks a wide range of crop plants from different families and is grown in a large variety of soils, in different seasons and under different climatic conditions of the agricultural regions. It has the ability to produce a large number of tiny seeds that maintain their vitality in the soil for decades [3]. Therefore, it significantly reduces the ability of using the field for potential sensitive hosts.

As a holoparasitic plant, *P. aegyptiaca* absorbs water, minerals and organic compounds from its host [4,5], redirecting the host solutes by creating a strong sink [6]. Despite the accumulated knowledge of many aspects of its biology, our knowledge about the metabolites that are transferred to the parasite and how the host affects the primary metabolic profile of the parasite is yet mostly unknown [7]. Furthermore, there is discussion in the scientific literature about the nature of the metabolites. One option is that most of the metabolites found in holoparasites are produced by the parasite, relying primarily on the organic carbon source transported from the hosts, while another option is that the parasite takes most of its metabolites from its host (e.g., [8,9,10,11]. Several lines of evidence support the first option. First, it was reported that the metabolic profiles of the parasite differ significantly from their hosts [9,12,13,14], suggesting that the parasites have the ability to change their metabolic profile. Second, the observation that several compounds that inhibit enzymes in amino acid biosynthetic pathways are able to kill broomrape [15,16] suggests that these enzymes are active in the parasite. Third, the parasite can form its own carotenoids, as recently reported [17].

Yet, many questions remain unanswered regarding the dependence of the parasite on the metabolites of their hosts [7]. Recently, in a study of the metabolic profiles of three main organs of the holoparasitic plant *Cuscuta campestris* (dodder) that developed on three different hosts, it was discovered that the levels of metabolites in the parasite differed significantly between the hosts [18]. This suggests that the parasites rely heavily on the host’s metabolites. However, changes in the metabolites’ contents between the organs of the parasite that developed on the same host demonstrate that the parasite can also self-regulate its own metabolites [18].

The goal of this study is to examine how 10 different hosts affect the primary metabolic profile of *P. aegyptiaca*. The knowledge obtained could help us distinguish between two options: whether most of the metabolites differ between the parasites that developed on the 10 hosts; or whether they have a similar metabolic profiling. If they exhibit different profiles, this indicates that they are heavily dependent on their host. However, if they show similarity, this suggests that they mostly produced their metabolites on their own. *P. aegyptiaca* was chosen since it infected broad crop hosts, and because information about its metabolites can form a basis for future manipulation that will reduce its infectivity in the fields.

## 2. Materials and Methods

### 2.1. Plant Materials and Sample Collection

*P. aegyptiaca* Pers. was collected from 10 different host species at different locations and on different dates (Table 1). For this study, we collected the parasite from 10 crop plants belonging to four families: (i) Brassicaceae [broccoli, red cabbage and white cabbage, all belonging to *Brassica oleracea*]; (ii) Apiaceae [carrot (*Daucus carota* subsp. *Sativus*), dill (*Anethum graveolens*) and fennel bulbs (*Foeniculum vulgare*)]; (iii) Solanaceae [potato (*Solanum tuberosum*), pepper (*Capsicum annuum*) and tomato (*Solanum lycopersicum*)]; and (iv) Fabaceae [chickpea *(Cicer arietinum*)]. The parasites were collected at different locations and on different dates (Table 1). All of the parasitic plants were collected at the same developmental stage, including the lower and upper stems of the parasite when the flower buds appeared [19]. The collected plants were transferred to the laboratory under cold conditions. In the laboratory, they were washed with cold water and dried with a towel and soft paper, cut into sections measuring about 3 cm and frozen immediately in liquid nitrogen, followed by lyophilization. The lyophilized plants were ground to fine powder by mortar and pestle and kept for further analysis at −20 °C.

### 2.2. Extraction of Primary Metabolites and Analysis Using Gas Chromatography-Mass Spectrometry (GC-MS)

The protocol to determine the primary metabolites is based on our previous studies [12,15,18,19,20]. Each of the broomrape samples was homogenized using a Restch MM 301 homogenizer. Twenty mg of the dry weight (DW) fine powder was mixed in 1000 µL of methanol/chloroform/double-distilled water (DDW) (2.5:1:1) at 4 °C. Norleucine (4.6 µL of 2 mg per mL) was added as an internal standard. After shortly vortexing the samples and 10 min of centrifugation at 20,000× *g* at 4 °C, 1000 µL of the supernatant was collected in a new tube. The lower phase was kept for fatty acid analysis. In this case, 300 µL DDW and 300 µL chloroform were added. Samples were treated and derivatized as previously described [12]. To determine retention time indices, 7 μL of n-alkane mixture (C12, C15, C19, C22, C28, C32 and C36, 2 μL mL, 1 μL of each in pyridine) was added. Acidic protons were then trimethylsilylated by adding 100 μL N-methyl-N-(trimethylsilyl) triflouroacetamide incubated with constant agitation for 30 min at 37 °C and transferred into GC-MS running vials. The injections to the GC-MS were made in random order. The total-ion-count method was used for the metabolic profiling and separation using the VF-5 ms capillary column (Agilent; 30 + 10 m EZ-guard, 0.25 mm i.d. and 0.25 mm thicknesses), while the single-ion mass method was used for soluble amino acid determination with an RXI-5-Sil MS capillary column (RESTEK; 30 m, 0.25 mm i.d. and 0.25 mm thickness) [12].

All analyses were carried out on a GC-MS system (Agilent 7890A) coupled with a mass selective detector (Agilent 5975c) and a Gerstel multipurpose sampler MPS2 [20]. Peak finding, peak integration and retention time correction were performed using the Agilent GC/MSD Productivity ChemStation package (http://www.agilent.com). Integrated peaks of the mass (m/z) fragments were normalized for sample dry weight and integral standard (norleucine) signal. The corresponding mass spectra and retention time indices were compared with standard substances and commercially available electron mass spectrum libraries from the National Institute of Standards and Technology (NIST) (http://www.nist.gov/) and Max Planck Institute for Plant Physiology, Golm, Germany (http://www.mpimp-golm.mpg.de/). Standard curves were generated for each amino acid, most of the sugars (such as sucrose, fructose, glucose and galactose), fatty acids, organic acid of the TCA cycle and polyols (such as mannitol and sorbitol). These standards and curves were used to identify and quantify the absolute levels of the different metabolites. All samples were also standardized relative to the sample dry weight used for extractions.

### 2.3. Total Soluble Protein Content Determination

For total soluble protein content determination, five mg dry-weight powder from the parasites was ground in 200 µL buffer phosphate pH = 7.8 with a protease inhibitor cocktail (Sigma Aldrich, St. Louis, MO, USA). After two centrifugation cycles (20,800× *g* for 5 min), the total soluble protein content was determined using a Bradford reagent (Bio-Rad) in three sample concentrations. Bovine serum albumin was used as a standard.

### 2.4. Total Phenolic Compound Content Determination

Twenty mg of lyophilized dried weight of broomrape powder was ground in 0.5 mL of water and centrifuged at 12,000× *g* for 20 min and supernatant was collected in fresh tubes. Ten µL of each of the extracted samples was loaded on a 96-well ELISA plate. In each well, 50 µL of 10% Folin-Ciocalteu reagent and 40 µL of 7.5% (*w*/*v*) Na_2_CO_3_ were added and mixed according to the colorimetric method that modified the Ben Nasr method for small volumes [21]. The plate was incubated for 40 min at 37 °C and read at 765 nm using a spectrophotometer (Infinite M200Pro). A standard curve was plotted using gallic acid as standard.

### 2.5. Statistical Analyses

The data represent the mean of four independent replications. Principal component analysis (PCA) and a heat map of GC-MS data were utilized using the MetaboAnalyst 5.0 comprehensive tool (http://metaboanalyst.ca/; [22]) with auto scaling (mean-centered and divided by the standard deviation of each variable) manipulations. Graphs were compiled using GraphPad Prism 5.01 scientific software (http://www.graphpad.com/). Biplot analyses were carried out using R Studio version 4.03 and graphics packages. Statistical significance was evaluated using JMP software version 8.0 (SAS Institute Inc., Cary, NC, USA). Significant differences between samples were calculated according to the Turkey–Kramer HSD test (*p* < 0.05). For the *p*-value adjustment we used the MetaboAnalyst that provides two methods of Fisher’s least significant difference method (Fisher’s LSD) and Tukey’s Honestly Significant Difference (Tukey’s HSD).

## 3. Results

### 3.1. Primary Metabolic Profiling Analysis Using GC-MS Reveals a Differential Metabolic Accumulation in *P. aegyptiaca* with Respect to the Host Plants

To explore the effect of the hosts on the primary metabolic profile of the parasite, *P. aegyptiaca* was collected from 10 hosts. All of the plants collected were at the same developmental stage that contained lower and upper stems and flower buds. The global primary metabolic profiling analysis and lipid profiling were performed according to GC-MS protocols [12,18,19,20] that previously enabled us to detect primary metabolites. The analysis revealed 58 annotated metabolites that comprised 16 amino acids, 12 sugars, 8 organic acids, 6 polyols, 5 fatty acids, 4 tricarboxylic acid cycle (TCA) intermediates, 4 sugar acids and three others (Supplementary Table S1). The *p*-value adjustment to each of the detected metabolites is given in Supplementary Table S2. The sugars also included one unannotated sugar (NA), which the GC-MS identified as sugar but could not indicate the right annotation according to the m/z ratio. All of the metabolites were identified by standards or by the retention index relative to alkane’s standards.

**Figure 1:**
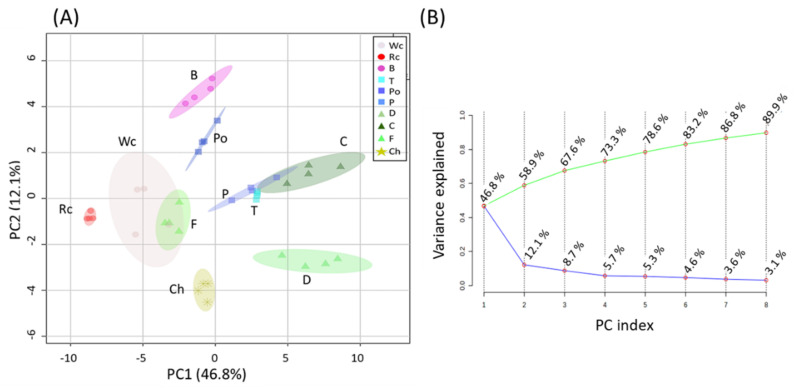
(**A**) Principal component analyses (PCA) applied to the levels of individual primary metabolites of *P. aegyptiaca* grown on 10 host plants according to the 58 primary metabolites. The data points are displayed as projections onto the two primary axes (eigenvectors). Variances explained by the first two components (PC1 and PC2) appear in parentheses. Host plants are designated as Broccoli (B), Red cabbage (Rc), White cabbage (Wc), Potato (Po), Pepper (P), Tomato (T), Carrot (C), Dill plant (D), Fennel bulbs (F) and Chickpea (Ch); (**B**) Display of the Scree plot of eight PCs. The green line on top shows the accumulated variance; the blue line below shows the variance explained by the individual PC.

First, a principal component analysis (PCA) was carried out to provide a general overview of the differences between the hosts (Figure 1A). Variances were explained by two components: PC1, which was responsible for 46.8%; and PC2, which gave a value of 12.1% of the variance, accounting for 58.9% of the total variance between broomrape on different hosts (Figure 1B). The results show that a relatively long distance was found between the parasites that developed on the different hosts. The distance between the samples existed even when the host belonged to the same plant family. Notably, fennel and white cabbage were relatively closer, as were tomato, pepper and carrot. This indicates that the hosts significantly affect the metabolic profile of *P. aegyptiaca*.

**Figure 2:**
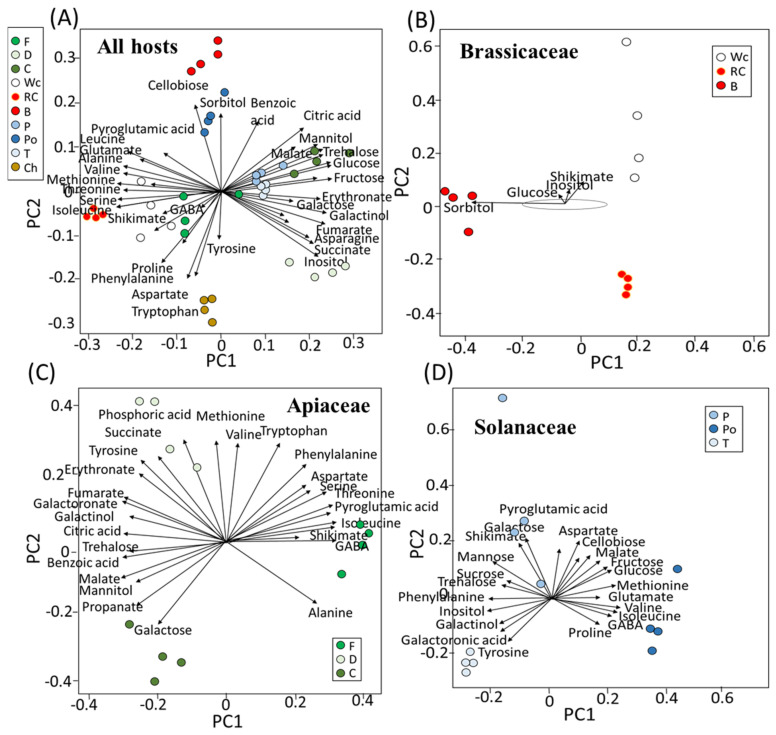
Biplot analyses. The length and direction of each vector represent the contribution of the metabolite to the PCA. (**A**) Metabolites from all 10 hosts and the vectors for the individual host family. Hosts that belong to the families of Brassicaceae (**B**), Apiaceae (**C**) and Solanaceae (**D**). Host plants are designated as Broccoli (B), Red cabbage (Rc), White cabbage (Wc), Potato (Po), Pepper (P), Tomato (T), Carrot (C), Dill plant (D), Fennel bulbs (F) and Chickpea (Ch).

To define the metabolites that mostly affect variance in the PCA, biplot analyses were made using R-based software (Figure 2). When all of the parasites that were grown on the 10 hosts were plotted together, cellobiose and sorbitol were shown to affect most of the variance in broccoli, while the variance in red and white cabbage was affected by shikimate, GABA, isoleucine, valine and methionine (Figure 2A). Sugars, such as glucose, fructose and trehalose, affected the variance in the parasites grown on carrot, while those developed on fennel were affected by inositol. Parasites that developed on dill were affected by shikimate, isoleucine, GABA and proline. Those grown on pepper and tomato were affected similarly to carrot, while potato was affected by sorbitol (Figure 2A). Aspartate, tyrosine and tryptophan affected the level of parasites developed on chickpea.

To explore if the parasites grown on the hosts from the same family had similar profiles, biplot analyses were performed on the three families that had three hosts (Figure 2B–D). Those developed on the hosts from Brassicaceae showed a significant distance (Figure 2B). Broccoli was affected by sorbitol and glucose, and white cabbage was affected by shikimate and inositol. In Apiaceae, the range of metabolites that affected the variance was greater. The parasites that developed on fennel were mainly affected by isoleucine, shikimate and GABA, while those that developed in dill were mainly affected by succinate and phosphoric acid. Carrot, however, was affected by galactose (Figure 2C). In Solanaceae, the main contributors for the variance in pepper were shikimate and galactose, which developed on potatoes affected by glucose, valine, GABA and isoleucine, and those that developed on tomato were mostly affected by tyrosine (Figure 2D).

**Figure 3:**
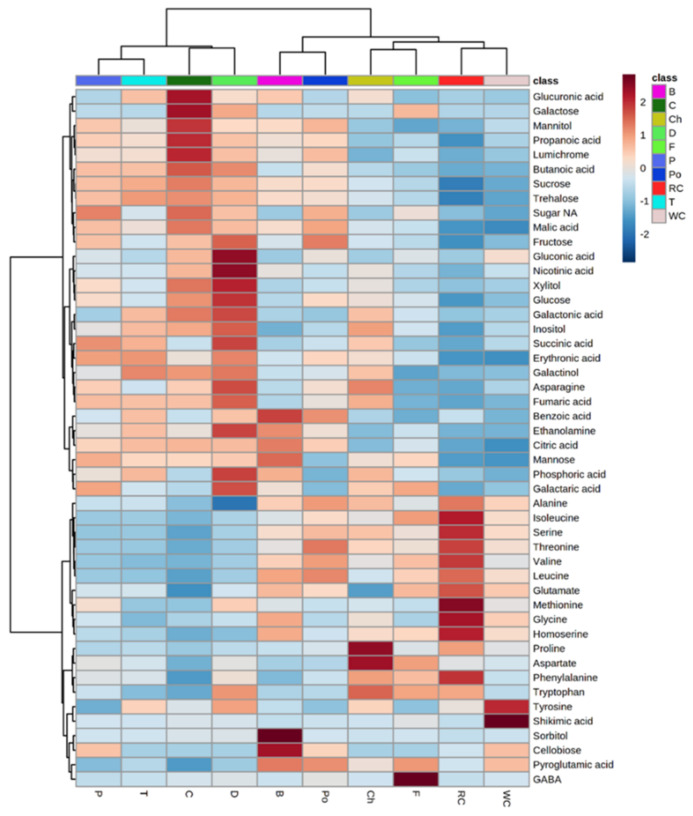
Heat-map analysis of the 58 primary metabolites detected by GC-MS. The data represent four replicates for each parasite that developed on each host plant. A total of 10 hosts were examined. Host plants are designated as Broccoli (B), Red cabbage (Rc), White cabbage (Wc), Potato (Po), Pepper (P), Tomato (T), Carrot (C), Dill plant (D), Fennel bulbs (F) and Chickpea (Ch).

To obtain a better resolution, each metabolite whose level had changed significantly between the different hosts was plotted separately using a heat-map analysis (Figure 3). The results show major differences in the metabolic profile of the parasite that developed on different hosts. We were not able to detect even a single metabolite whose level was relatively constant between the various hosts. The upper dendogram shows two clusters (Figure 3). The left contains the parasites collected from dill, carrot, tomato and pepper that had higher levels of organic acids (including those of the TCA cycle), several sugars and polyols compared to those developed on other hosts (Figure 3). However, those developed on broccoli, potato, chickpea, fennel, red cabbage and white cabbage had high levels of most of the amino acids (Figure 3). These observations strongly support the hypothesis that the hosts significantly affect the levels of metabolites in *P. aegyptiaca* plants. The left dendogram shows two clusters: the upper one contains the sugars and organic acids; the lower one contains most of the amino acids (Figure 3). Based on these results, we got the impression that there are two groups of hosts: those that cause accumulation of amino acids in the parasite and others that cause the accumulation of sugars and organic acids.

**Figure 4:**
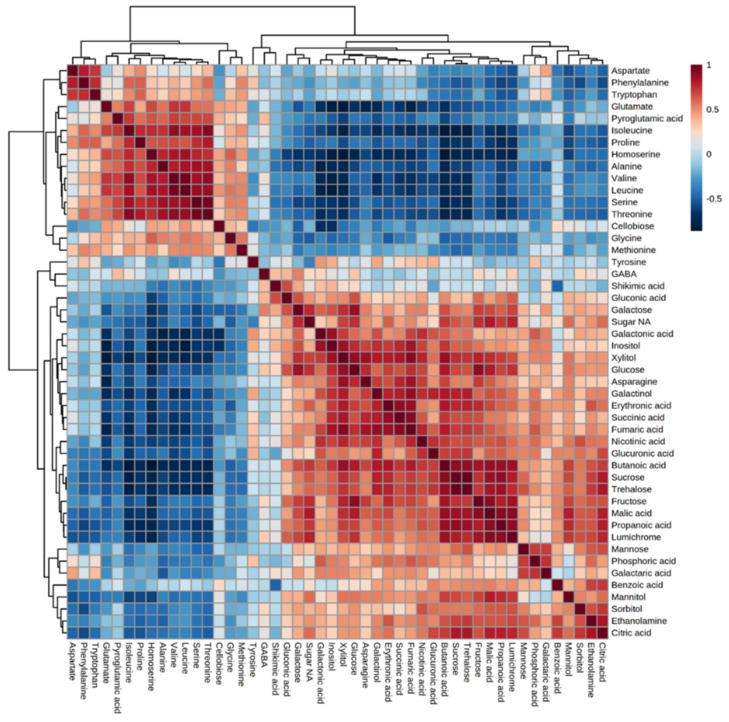
Correlation matrix among the 58 metabolites detected in the parasites that developed on the 10 different hosts using Pearson correlation coefficients. Each data point is the average of four biological replicates. Dendrograms are shown on the top and left of the correlation matrix, indicating the clustering of positive and negative correlations. Red and blue colors indicate positive and negative correlations, respectively.

This metabolic phenotype could result from positive or negative interactions between the biosynthesis pathways, leading to these metabolites. To gain more knowledge about the relationship between the metabolites detected, a correlation matrix analysis was made between all of the metabolites detected (Figure 4). The results showed two clusters that correlated negatively: the cluster that contained all of the amino acids (except for tyrosine) and the second that included the other metabolites. A strong positive correlation was found in the branched chain amino acids (BCAA)—valine, leucine and isoleucine—as well as with threonine, serine, proline and alanine, most of which accumulated under abiotic stress [23]. The biosynthesis pathways of BCAA, threonine and serine were found to be strongly connected [24].

**Figure 5:**
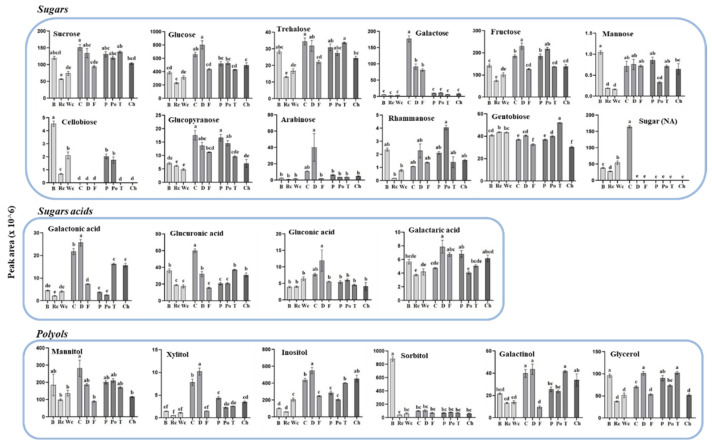
The levels of individual primary metabolites of sugars, sugar acids and polyols of *P. aegyptiaca* grown on 10 host plants as detected by using GC-MS. Values are relative peak areas normalized to the norleucine internal standard. The Y axis represents the area of relative m/z response of each metabolite following normalization to the norleucine internal standard and the X axis represents the host plants designated as Broccoli (B), Red cabbage (Rc), White cabbage (Wc), Carrot (C), Dill plant (D), Fennel bulbs (F), Pepper (P), Potato (Po), Tomato (T) and Chickpea (Ch). Data shown are means ± SE of four replicates for each plant type. Significance is calculated according to the Turkey Kramer HSD test (*p* < 0.05) and is denoted by different small letters. NA—non-annotated sugar.

**Figure 6:**
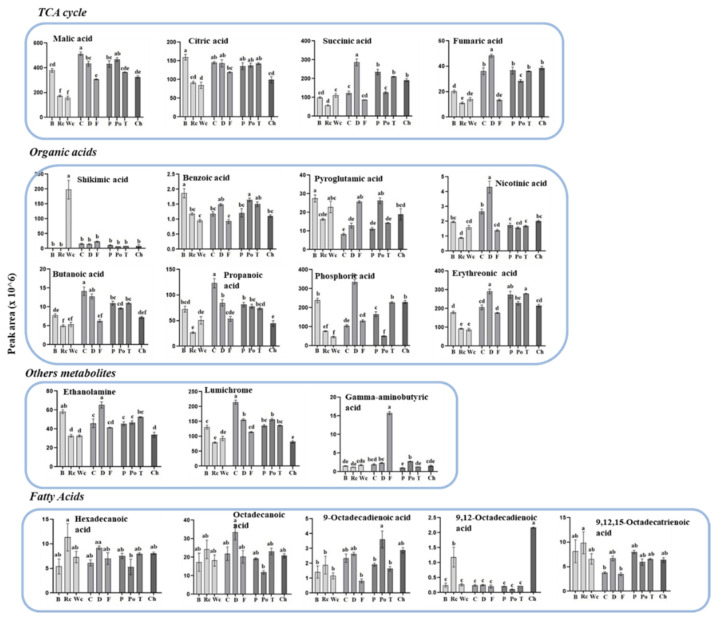
The levels of individual primary metabolites of organic acids, fatty acids and other acids of *P. aegyptiaca* grown on 10 host plants as detected by using GC-MS. Values are relative peak areas normalized to the norleucine internal standard. The Y axis represents the area of relative m/z response of each metabolite following normalization to the norleucine internal standard and the X axis represents the host plants designated as Broccoli (B), Red cabbage (Rc), White cabbage (Wc), Carrot (C), Dill plant (D), Fennel bulbs (F), Pepper (P), Potato (Po), Tomato (T) and Chickpea (Ch). Data shown are means ± SE of four replicates for each plant type. Significance is calculated according to the Turkey Kramer HSD test (*p* < 0.05) and is denoted by different lowercase letters.

**Figure 7:**
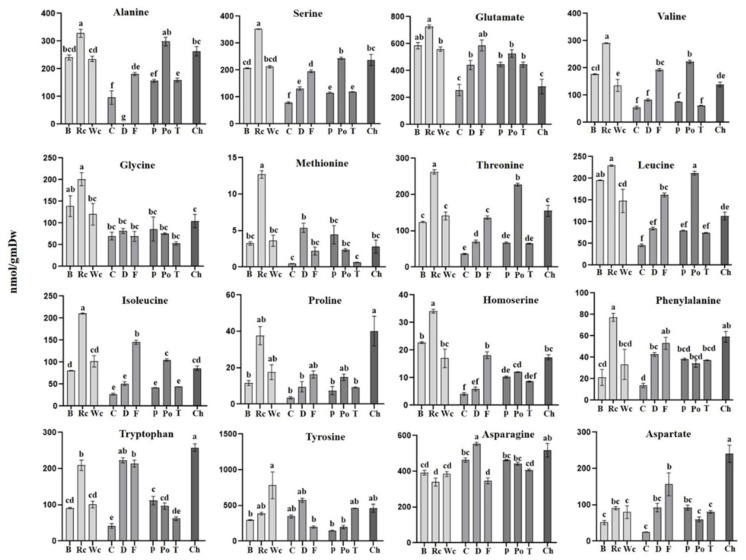
The levels of individual amino acids of *P. aegyptiaca* grown on 10 host plants as detected by using GC-MS. Values are normalized to the norleucine internal standard. Levels of different amino acids accumulated in *P. aegyptiaca* grown on 10 host plants collected from Broccoli (B), Red cabbage (Rc), White cabbage (Wc), Carrot (C), Dill plant (D), Fennel bulbs (F), Pepper (P), Potato (Po), Tomato (T) and Chickpea (Ch). Data shown are means ± SE of four replicates for each plant type. Significance is calculated according to the Turkey Kramer HSD test (*p* < 0.05) and is denoted by different lowercase letters.

To gain more information about the differences between the accumulation of these two clusters and to define more accurately how the host affects each of the metabolites detected in the parasite, the individual metabolites were plotted separately and statistical tests were used to differentiate between levels in the parasite that developed in each of the 10 hosts. The results (Figure 5, Figure 6 and Figure 7) show that the hosts significantly affected the levels of metabolites in the parasites. In most of the metabolites, significant differences were found, even within members of the same family. When sugars were detected, it was found that *P. aegyptiaca* that developed on carrot and dill, both belonging to the Apiaceae family, exhibited the highest levels of sucrose, glucose, fructose and trehalose; carrot also exhibited high levels of galactose, glucopyaranose and sugar (NA). From the Brassicaceae family, broccoli had the highest level of sugars (7 out of 12), whereas from the Solanaceae family, potato showed the highest content of rhammanose and tomato showed the highest content of gentobiose compared to the other parasites that developed on the different hosts (Figure 5). When sugar acids were measured in the parasites, the highest levels of galactonic acid, glucorunic acid and gluconic acid were detected from those developed on carrot and dill (Figure 5).

The three Brassicaceae plants exhibited relatively low levels of the four metabolites detected in the TCA cycle compared to the other parasitic plants (Figure 6). The parasites that developed on carrot and dill tended to have the highest accumulation of most of the organic acids (six out of nine). They also showed high levels in four out of five of the polyols detected. *P. aegyptiaca* that developed on broccoli had the highest levels of pyroglutamic acid, benzoic acid and sorbitol. Significant differences were also detected in the levels of fatty acids and three other metabolites (Figure 6).

The results of the amino acid analysis (Figure 7) showed that the hosts significantly affected their levels in the parasites. In general, red cabbage, as a host, provided a significantly higher level of most of the amino acids, such as BCAA (isoleucine and valine), aromatic amino acid phenylalanine, as well as glycine, serine, methionine and alanine compared to *P. aegyptiaca* collected from the other hosts (Figure 4). High levels of BCAA (6- to 40-fold) were also detected in other holoparasitic plants that were compared to other parasitic plants from the groups of hemiparasitic and mycoheterotrophic plants [25]. The parasite that developed on chickpea had the highest contents of proline and tryptophan. From the three Apiaceae members, fennel had the highest level of amino acids (11 out of 16 detected amino acids), while from the Solanaceae, potato exhibited the highest level (9 out of 16). In a very general view, parasitic plants developed on hosts from Brassicaceae apparently had higher levels of amino acids but relatively low levels of sugars, while those developed on hosts from Apiaceae and Solanaceae tended to have more sugars and organic acids and less amino acids compared to those grown on Brassicaceae (as shown in Figure 3).

Taken together, the results indicate that the hosts significantly affected the levels of metabolite accumulation in parasitic plants.

### 3.2. Determination of Total Soluble Proteins and Total Phenolic Compounds

**Figure 8:**
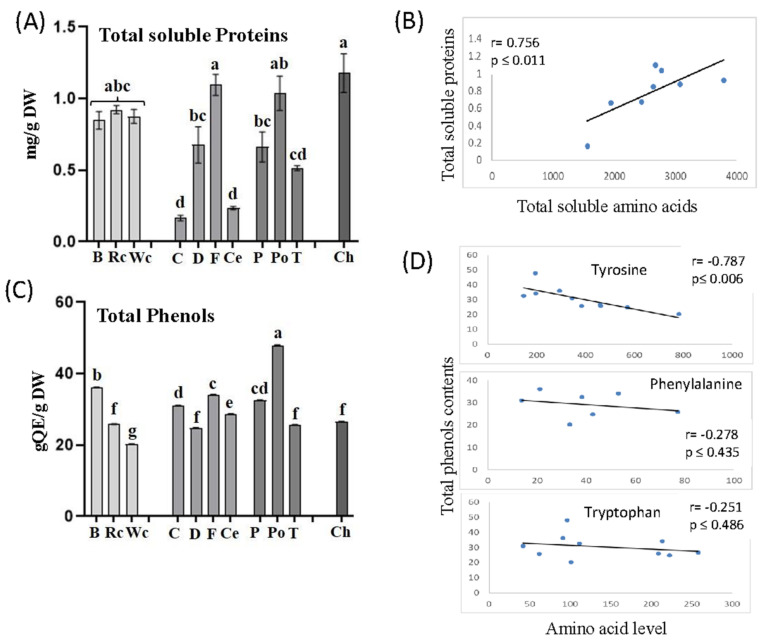
The total soluble proteins (**A**) and total phenol contents (**C**) in the parasites that developed on 10 different hosts. Pearson’s correlation coefficient analyses between total soluble amino acids and total soluble proteins (**B**) or between the levels of three aromatic amino acids and total phenol contents (**D**). Total protein contents in the albumin fraction are measured using the Bradford assay; total phenol contents are represented as mg quercetin equivalents (QE) per mg of dry weight (DW). All data shown are means ± SE of four replicates. The significance is calculated according to the Tukey–Kramer HSD test (*p* < 0.05) and is identified by different lowercase letters.

*P. aegyptiaca* that developed on chickpea, red and white cabbage contained relatively high levels of amino acids. To determine if these high levels affect total soluble proteins in *P. aegyptiaca*, we carried out a Bradford analysis on soluble protein fractions that were extracted from *P. aegyptiaca* that developed on the 10 hosts. The analyses revealed that the protein content indeed tended to be highest in parasites collected from chickpea, followed by fennel and potato, whereas the lowest amount of protein was accumulated in parasites developed on carrot. The parasites that developed on three members of the Brassicaceae family (broccoli, red and white cabbage) also exhibited significantly higher levels of soluble protein compared to dill, pepper and tomato (Figure 8A). To further examine these interactions, we calculated the correlation coefficient values (r) between total soluble amino acids and total soluble proteins obtained from the parasites that developed on 10 different hosts. A significant positive correlation was found between the two variables (r = 0.756, *p* ≤ 0.01), implying a strong impact of soluble amino acids on soluble protein contents in *P. aegyptiaca* (Figure 8B).

The relatively high levels of aromatic amino acids in the parasitic plants that developed on the different hosts suggest that they can influence the synthesis of phenolic compounds. Such a link was reported in *P. aegyptiaca*, which showed the highest level of aromatic amino acids and total phenolic compounds in flowers and flower buds compared to other organs of the parasitic plant [19]. To verify this assumption, the levels of total soluble phenolic compounds were examined in the parasites that developed on the 10 hosts. The results showed that the highest total phenolic content was found in *P. aegyptiaca* collected from potato followed by broccoli, while the lowest was detected in those developed on white cabbage (Figure 8C). Correlation coefficient values (r) were calculated to examine the link between the levels of aromatic acids and total phenols. A significant negative correlation was found between tyrosine and phenol content (r = −0.787, *p* ≤ 0.006). The two other aromatic amino acids tended to have negative but insignificant correlations with the phenols. This suggests that these three amino acids were used to form the phenols in the parasite (Figure 8D).

## 4. Discussion

The first aim of the current study was to determine the effect of 10 hosts on the primary metabolic profile of *P. aegyptiaca*. We assume that if a parasite is taken from its host, the main sugars (sucrose, glucose and fructose) and basic nitrogen compounds are used to synthesize its own metabolites, the metabolic profile of the parasites that develops on the 10 hosts will be relatively similar. This assumption was also proposed for other holoparasites [26,27,28]. This assumption is also based on genome analysis, showing that the holoparasites have genes that can form most of their metabolites [29].

However, if the parasite absorbs different metabolites from the host, the parasite’s metabolic profile will vary significantly between different hosts. The results in the current study showed that *P. aegyptiaca* that developed on the different hosts had significantly different contents of primary metabolites (Figure 1, Figure 2, Figure 3, Figure 4, Figure 5, Figure 6 and Figure 7). These differences might result from the different hosts, but also from the different environmental conditions that prevailed when the hosts and the parasite were developed. The annual seasons could also affect the metabolic profiles, as well as the soil type and the health and developmental stage of the host. The observation that the parasite collected from potato is at a greater distance from those grown on tomato and pepper (that belong to Solanaceae) in the PCA analysis (Figure 1) might be attributed to the fact that potato was collected from other habitats in a different season than the two others. However, the observation that significant differences occur in the metabolite contents between the parasites that harvest on the same day from the neighboring fields suggests that the type of host is a dominant factor leading to these results. This is the case for the groups of tomato and pepper; white and red cabbage, broccoli and fennel; and dill and chickpea. These three groups separate well on the PCA and biplot in PCs 1–2 (Figure 1 and Figure 2).

The results of the current study suggest that the parasite depends heavily on its host’s metabolites. Support of this assumption also comes from the finding that *P. aegyptiaca*, in general, accumulates more sugars (when developed on members of the Apiaceae and Solanaceae families), while having a low level of amino acids. However, when the parasite developed on hosts of the Brassicaceae family, it had high amino acids and low sugars. These findings are in accordance with previous results, suggesting that the host significantly affects the metabolite levels of *P. aegyptiaca* [9] and *C. campestris* [18]. The results also suggest that *P. aegyptiaca* transports nutrients from its hosts, such as carbohydrates and nitrogen compounds, but also many other primary and secondary metabolite compounds, as previously reported [8,9,10].

However, the observation that the levels of most of the metabolites in holoparasites differ significantly from their hosts [9,12,30] implies that the parasites can accumulate and/or synthesize metabolites in different ways than their hosts; therefore, they most probably used their genes to self-regulate their contents. The finding that the holoparasite *C. campestris* has a different metabolic profile in three different organs that developed on the same parasitic plant strongly indicates that the parasite can self-regulate its own metabolites. In addition, the observation that the metabolic composition significantly changed during the developmental stages of *P. aegyptiaca* and *P. ramose* [9,19,31] and in *Cuscuta japonica* [32] also suggests that the parasites can self-regulate their metabolites, depending on their developmental stage. One good example of this self-producing metabolite is mannitol. Parasites of the Orobanchaceae and Phelipanche families convert hexoses into mannitol in order to increase sink strength, while only traces of mannitol can be detected in their hosts [12,31,33]. Mannitol can accumulate to a high level within the cells without disrupting their function [34], which could be about 40-fold in *P. aegyptiaca* compared to its host roots [12]. Another example is the uptake and accumulation of alkaloids by *Cuscuta*, while the hosts have only traces of these alkaloids [35,36]. Indications of the presence of functional pathways in *P. aegyptiaca* were recently noted after studying the effects of herbicides targeting the specific enzymes in the biosynthesis pathway aromatic and BCAA amino acids [15,16]. These results showed that the parasite has sets of enzymes that are significantly more sensitive to these herbicides than their hosts are.

The debate about the nature of the compounds derived from the host is still ongoing in the scientific literature, and evidence of both options (transporting their needs from the host or self-regulating their metabolites) has been reported. The significant dependence on the hosts was shown by measuring the dry weight, stem length and number of attachment sites of *C. campestris* that developed on three hosts [33]. A more recent study that measured sugars, sugar alcohols and carboxylic acids before and after the attachment of *P. aegyptiaca* to the hosts reported that *P. aegyptiaca* relies on the hosts for different carbon resources and other primary metabolites [9]. However, it also reported that the parasite’s metabolite profiles reflected more the developmental stage of the parasite than the host’s resource availability [9]. Questions also remain as to whether the parasite can control what is transferred from its host or whether it is simply connected by a “pipe” to the host and, thus, lacks the ability to control the metabolites that are transferred. It was indicated that *C. campestris* and *C. japonica* lack the ability to select specific metabolites from their hosts [27,37,38]. Upon examining the secondary metabolites of *C. reflexa* in the parasite that developed on two different hosts, it was revealed that specific compounds that synthesize in each host can be detected in the parasite [39]. Further, significant differences were found in the contents of flavonoids identified in *C. reflexa* plants that developed on five different hosts [40]. This variation confirms that some metabolites in the parasite were derived from the host with minimum selection.

Two findings obtained in the current study are not trivial and require further examination. First, the biplot analysis showed that sugars, shikimate phosphoric acid and polyols are the main factors influencing the variance in the parasite’s metabolites between the different hosts. This is similar to the results obtained from the biplot for *C. campestris* that developed on different hosts [18]. Second, the correlation test between the different metabolites showed a cluster in which different amino acids were strongly correlated and another cluster that contained other metabolites, mainly sugars and organic acids. The strong correlation between the amino acids suggests that the parasite has control factors that lead to their accumulation and can even control their synthesis in the parasite. In the future, it would be interesting to distinguish between the two possibilities.

The high level of amino acids in the parasite [12] also suggests a high level of proteins in this parasite. To determine this link, a correlation test was carried out that showed a significant positive correlation. When total soluble protein levels were measured in different parasites that were hemiparasitic, holoparasitic or mycoheterotrophic, it was detected that the holoparasitic species was characterized by increased levels of total soluble proteins [25]. In addition to soluble proteins, it was reported that species of Orobanche and other holoparasitic plants contained very high levels of starch, an array of minor sugars (palatinose, turanose, arabinose), the organic acid shikimate and intermediate levels of the sugar alcohol of myo-inositol [12,25].

This metabolic study is a first step towards understanding the ability of the parasite to accumulate and/or produce its own metabolites. Future studies should focus on assaying the metabolic profile of the parasites grown on the same host in different habitats to reveal the effect of the environmental conditions. Future studies should focus on examining the levels of the metabolites in the hosts and in its attached parasite in different developmental stages, to understand which metabolites are derived from the hosts and accumulate in the parasite. This analysis will also help determine the metabolic similarity between the host and the parasite plants.

## 5. Conclusions

This study shows that hosts significantly affected the levels of the primary metabolites in *P. aegyptiaca*. Thus, the results suggest that *P. aegyptiaca* does not produce most of its metabolites on its own from sugars that it absorbs from the hosts, but rather it takes many more metabolites from its host. The absorption led to major differences in the metabolic profiles between the parasites that developed on the different hosts. Since the changes in the metabolic profiles between the parasites are so significant, we assume that the metabolites flux passively from the host. The results show that the hosts can be divided into two groups: one group whose parasites accumulated sugars and organic acids, which mainly belong to Apiaceae and Solanaceae families; and the other group that comprises hosts from Brassicaceae and Fabaceae families whose parasites accumulated mainly amino acids (Figure 3). Notably, the correlation matrix analysis showed a negative correlation between amino acids and the other metabolites (Figure 5). Despite these changes, the biplot analysis (Figure 2) showed that sugars, shikimate, phosphoric acid and polyols are the main factors influencing the variance of the parasite’s metabolites between the different hosts.

The results of this study enhance our knowledge about the metabolic relationship between the hosts and *P. aegyptiaca*. This accumulated knowledge could potentially lead to ways to reduce the growth of the parasites, and thus eliminate its tremendous damage to crop plants.

## Figures and Tables

**Table 1:** The locations and dates of the parasites collected from the different hosts.

**Hosts**	**Short Name**	**L** **ocations**	**Date**
Potato	Po	South Hula Valley	26 December 2018
Tomato	T	Beit She’an Valley	12 June 2019
Paprika pepper	P	Beit She’an Valley	12 June 2019
Broccoli	B	Jezreel Valley	11 January 2019
White cabbage	Wc	Jezreel Valley	11 January 2019
Red cabbage	Rc	Jezreel Valley	11 January 2019
Fennel	F	Jezreel Valley	11 January 2019
Dill	D	Beit She’an Valley	21 May 2019
Carrot	C	North Hula Valley	14 April 2019
Chickpea	CH	Beit She’an Valley	21 May 2019

## Data Availability

Data is contained within the article or Supplementary Materials.

## Supplementary Materials

The following supporting information can be downloaded at: https://www.mdpi.com/article/10.3390/metabo12121195/s1, Table S1: The levels of individual primary metabolites of *P. aegyptiaca* that developed on ten hosts, as detected by using GC-MS; Table S2: The f and *p* values as well as the FDR for each of the detected metabolites.
